# Post-reconstruction enhancement of [^18^F]FDG PET images with a convolutional neural network

**DOI:** 10.1186/s13550-021-00788-5

**Published:** 2021-05-11

**Authors:** John Ly, David Minarik, Jonas Jögi, Per Wollmer, Elin Trägårdh

**Affiliations:** 1 Department of Radiology, Kristianstad Hospital, Kristianstad, Sweden; 2 Department of Translational Medicine, Lund University, Malmö, Sweden; 3 Radiation Physics, Skåne University Hospital and Lund University, Lund, Malmö, Sweden; 4 Clinical Physiology and Nuclear Medicine, Skåne University Hospital and Lund University, Malmö, Sweden; 5 Wallenberg Center for Molecular Medicine, Lund University, Lund, Sweden

**Keywords:** Cancer, Artificial intelligence, PET, Image quality

## Abstract

**Background:**

The aim of the study was to develop and test an artificial intelligence (AI)-based method to improve the quality of [^18^F]fluorodeoxyglucose (FDG) positron emission tomography (PET) images.

**Methods:**

A convolutional neural network (CNN) was trained by using pairs of excellent (acquisition time of 6 min/bed position) and standard (acquisition time of 1.5 min/bed position) or sub-standard (acquisition time of 1 min/bed position) images from 72 patients. A test group of 25 patients was used to validate the CNN qualitatively and quantitatively with 5 different image sets per patient: 4 min/bed position, 1.5 min/bed position with and without CNN, and 1 min/bed position with and without CNN.

**Results:**

Difference in hotspot maximum or peak standardized uptake value between the standard 1.5 min and 1.5 min CNN images fell short of significance. Coefficient of variation, the noise level, was lower in the CNN-enhanced images compared with standard 1 min and 1.5 min images. Physicians ranked the 1.5 min CNN and the 4 min images highest regarding image quality (noise and contrast) and the standard 1 min images lowest.

**Conclusions:**

AI can enhance [^18^F]FDG-PET images to reduce noise and increase contrast compared with standard images whilst keeping SUV_max/peak_ stability. There were significant differences in scoring between the 1.5 min and 1.5 min CNN image sets in all comparisons, the latter had higher scores in noise and contrast. Furthermore, difference in SUV_max_ and SUV_peak_ fell short of significance for that pair. The improved image quality can potentially be used either to provide better images to the nuclear medicine physicians or to reduce acquisition time/administered activity.

## Background

Positron emission tomography (PET) images are inherently noisy, and with a low spatial resolution, due to the specifics of the imaging process and the ill-posed tomographic reconstruction problem. Different reconstruction algorithms exist to improve image quality. For example, the block-sequential regularization expectation maximization algorithm (BSREM) [1, 2], commercially known as Q.Clear (GE Healthcare, Milwaukee, WI, USA) [3], has been developed. The algorithm has been shown to increase lesion detectability and the quantitative accuracy of standardized uptake value (SUV), particularly in small lesions, compared with standard ordered subset expectation maximization (OSEM) algorithms [2, 4, 5]. BSREM suppresses noise via a penalty factor beta (β), which allows for more iterations while keeping the noise level low. BSREM has been optimized for several different radiopharmaceuticals [6–9]. For [^18^F]fluorodeoxyglucose (FDG) PET, a β of 500 has been shown to be optimal if a scan time of 1.5 min per bed position with an administered activity of 4 MBq/kg is used. A lower acquisition time, i.e. 1 min/bed position gives images of too poor quality, given the same amount of activity [7].

Artificial intelligence (AI) is believed to transform radiology and nuclear medicine in the future [10]. Convolutional neural networks (CNN), an AI algorithm, have been shown to work well in improving image quality in planar nuclear medicine [11]. AI algorithms for image enhancement [11], segmentation [12–14], classification and prognostication [15] have been published. For example, standard-dose computed tomography (CT) [16] and PET images [17] has been derived from low-dose data using CNNs.

Modern nuclear medicine departments aim at increasing patient throughput due to a high demand, which makes short acquisition time important. However, this will decrease image quality unless the administered activity is increased. The aim of this study was therefore to test if image quality of BSREM reconstructed images obtained with short and standard acquisition times can be improved using a CNN trained on images acquired with a long acquisition time.

## Method

### Patients and imaging

Ninety-seven patients referred for clinical [^18^F]FDG PET-CT at Skåne University Hospital, Malmö or Lund, were included in the study. Seventy-two of the patients were used for training the CNN (included December 2019 to March 2020) and a separate set of 25 patients (included April to June 2018) were used to evaluate the method. All patients underwent an intravenous injection of 4 MBq/kg body weight of [^18^F]FDG after at least 4 h fasting and at a glucose level ≤ 10 mM. Four Discovery MI (GE Healthcare, Milwaukee, WI, USA) PET-CT systems, each with four detector rings, were used for image acquisition. Imaging was performed 60 min after administration, and the patients were scanned from the inguinal region to the base of the skull. The patients in the training set were scanned with 5–7 bed positions, depending on the length of the patient. The acquisition time for one of the bed positions was 6 min (different bed position for different patients) and 1.5 min for the others. The images of the patients in the test group were acquired with a time per bed position of 4 min for all bed positions and stored in list-mode.

The PET-images were reconstructed using a BSREM algorithm including time-of-flight and point spread function with a 256 × 256 matrix (pixel size 2.7 × 2.7 mm^2^, slice thickness 2.8 mm). CT images were acquired for attenuation correction and anatomic correlation of the PET images. A diagnostic CT with intravenous and oral contrast or a low-dose CT without contrast was performed.

### CNN

**Fig. 1:**
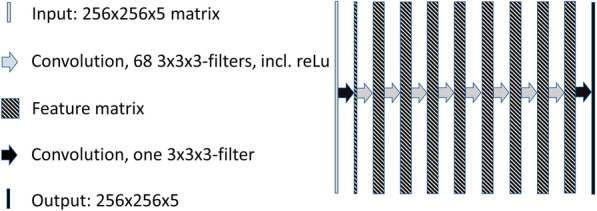
Diagram of the denoising CNN architecture. A mean squared error loss and a stochastic gradient descent optimizer was used

A denoising CNN suitable for image enhancement as proposed by Zhang et al. [18] was implemented using Matlab (Mathworks Inc. Natick, Massachusetts). In our adaptation of Zhang’s model, we have changed the number of convolution layers to 10 and used a 256 × 256 × 5 (3D) matrix input. Each convolution layer consists of 68 3 × 3 × 3 filters except the first and last layer which consist of one 3 × 3 × 3 filter. As in the proposed CNN by Zhang et al., batch normalisation was used in order to speed up the training process and improve the denoising performance. Pooling layers were not used in order to benefit from a larger receptive field where contextual information can be used to improve the denoising. A linear rectifier was used after each convolution layer except the last. A mean squared error loss and a stochastic gradient descent optimizer was used. An illustration of the current paper’s adapted CNN is shown in Fig. 1.

**Fig. 2:**
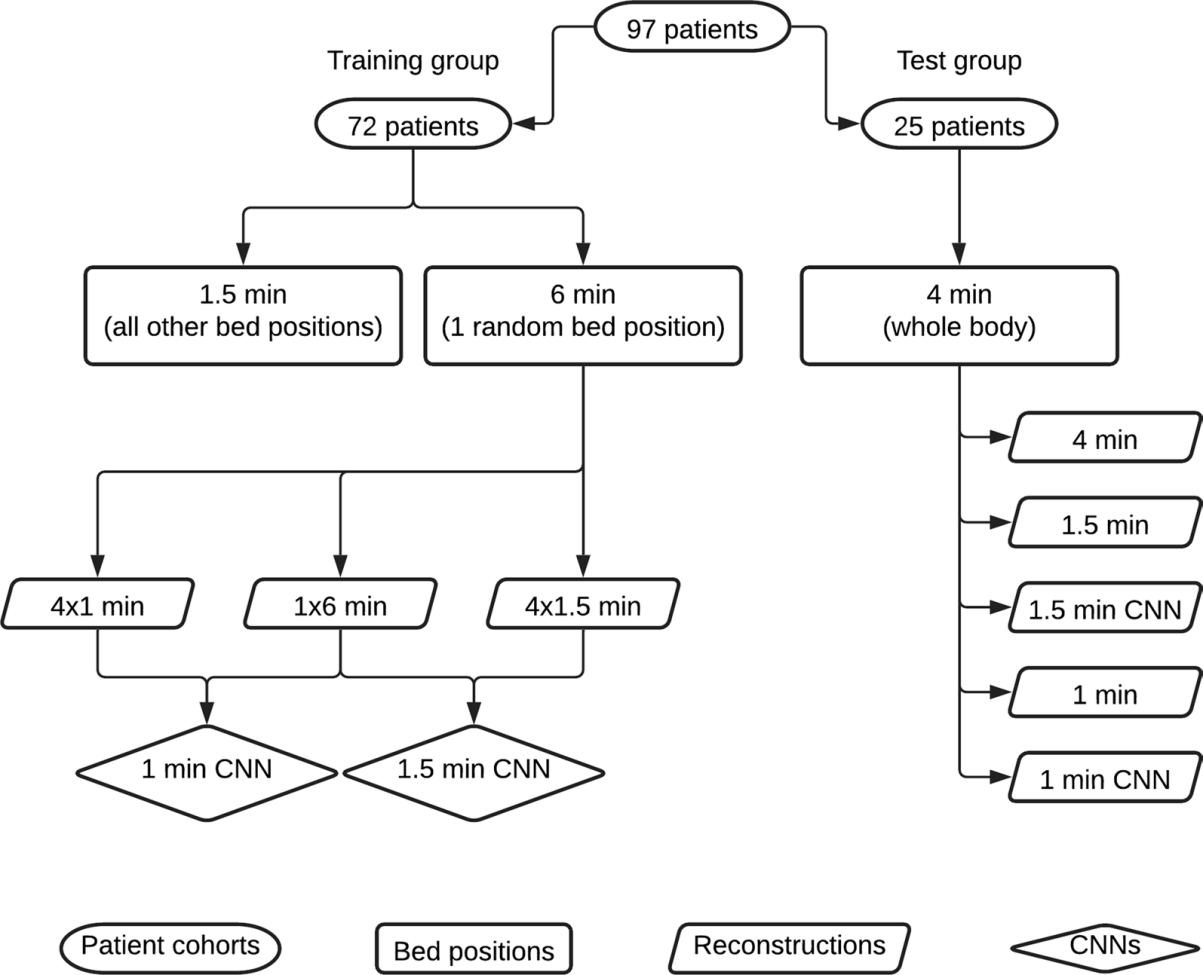
Diagram showing the scan time per bed positions and the derived BSREM reconstructions. The training group produces two networks (1 min CNN and 1.5 min CNN), these are applied on whole body reconstructions (1 min CNN and 1.5 min CNN) in the test group

List mode data from the bed position with a 6 min scan time were extracted from each of the 72 patients in the training group. List mode permits image reconstruction event-by-event from a chosen starting point and interval. From the list mode data, 1 image set for 6 min and 4 image sets for 1 (first, second, third and fourth minute) and 1.5 min (6 min divided in 4 intervals) were reconstructed with the BSREM algorithm, respectively. For the 1 min and 1.5 min images, a β of 500 was used, and a β of 200 was used for the 6 min image. Each reconstruction yielded 71 slices which were cropped in the beginning and the end, leaving only 50 of the centremost slices. Each 50-slice reconstruction were further divided into 10 subsets, each comprising a 3D volume sized 256 × 256 × 5, to match the input of the CNN. Furthermore, to reduce overfitting data augmentation was performed; each reconstruction (1 min and 1.5 min) was randomly resized, sheared and flipped in two dimensions, resulting in 5 additional samples. Two sets of training pairs were composed, pairs with 1 min and 6 min images and pairs with 1.5 min and 6 min images. The total training pairs for each of the two training sets were (subsets × patients × reconstructions × samples) 10 × 72 × 4 × 6 = 17.280. Two networks were trained: one for images acquired with 1 min and the other for 1.5 min/bed position. Figure 2 shows an overview how the training group and test group are set up. Table 1 shows an overview of the image sets from the training group.

**Table 1:** Overview of the image sets in the training group

Training group	
Image set	Beta
6 min	200
1.5 min	500
1 min	500

Two CNNs were trained using subset pairs, one consisting of 6 min and 1.5 min and the other was 6 min and 1 min

### Image analysis

25 patients (test group), each with 5 different whole-body image sets (Table 2), resulting in a total of 125 examinations, were evaluated; β500 1 min with and without CNN enhancement, β500 1.5 min with and without CNN enhancement and β300 4 min without CNN enhancement. In our department, β500 1.5 min is used clinically. The choice of β500 1 min + /− CNN is because it is a natural step if one wishes to decrease acquisition time to increase the throughput in the department. β300 4 min was considered as the gold standard image for reference. Image sets will henceforth be written without the beta-value for brevity.

**Table 2:** Overview of the whole-body image sets in the test group

Test group	
Image set	Beta
4 min	300
1.5 min	500
1.5 min CNN	500
1 min	500
1 min CNN	500

Each patient in the test group (n = 25) had 5 image sets, in total there were 125 examinations for readers to score

### Quantitative analysis

The coefficient of variation (COV), considered as an objective measure of noise, was calculated from regions of interests (ROIs) drawn in the liver on transaxial images using Hermes 2.0.0 (Hermes Medical Solutions, Stockholm, Sweden). Three ROIs with a diameter of 6 cm were drawn in subsequent transaxial slices with one image in-between, and the measurements were averaged. None of the ROIs were placed where liver metastases or large vessels were seen. The ROIs were drawn in the 4 min image set and copied to the other image sets. The COV was calculated as the ratio between the SUV_standard deviation (SD)_ and the SUV_mean_, thus a lower value indicates less noise.

The lesion SUV_max/peak_ were calculated from a VOI defined over a lesion. Three small-middle sized hotspots (pathologic lesions or physiologic uptake) per patient were selected to get a large range of SUVs. SUV_max_ and SUV_peak_ (SUV_mean_ in a 1 cm^3^ volume sphere) were calculated for all lesions.

### Qualitative analysis

Firstly, a nuclear medicine specialist evaluated all image sets per patient side-by-side in all anatomic planes to ensure the CNN didn’t add or subtract anything relevant from the images.

Secondly, two nuclear medicine specialists, including the previously mentioned and a radiology resident was presented with a blinded list consisting the 125 examinations in random order. Noise and contrast levels were recorded for each examination on a 5-point scale.

A training/calibration session was held to establish a baseline for noise and contrast levels. Images from patients not included in the test group with previously 5 mentioned image sets series were displayed side-by-side. 3 points in noise and contrast was established as baseline for standard 1.5 min (the clinically used images). For noise, 5 = “very little noise”, 4 = “slightly less noise compared with baseline”, 2 = “slightly increased noise compared with baseline”, 1 = markedly increased noise”. For contrast, 5 = “very good contrast”, 4 = “slightly increased contrast compared with baseline”, 2 = “slightly decreased contrast compared with baseline” and 1 = “markedly decreased contrast”.

Assessment of examinations was performed on dedicated workstations using Hermes 2.0.0.

### Statistical analysis

Friedman’s test was used to compare quantitative data for SUV_max_, SUV_peak_ and COV measurements in all 5 image sets (4 min, 1.5 min + /− CNN, 1 min + /− CNN), significant *p* value was set at *p* < 0.05. Post-hoc analysis with Wilcoxon signed-rank test was conducted with a Bonferroni correction applied, resulting in a significance level set at *p* < 0.005. Since there were 5 image sets, each measurement group resulted in 10 comparisons and thus significant *p* value was calculated as 0.05/10 = 0.005.

Kruskal–Wallis test was applied to each investigator’s results for the investigation of potential difference between the groups. Post-hoc testing with Mann–Whitney U tests were performed on all pairwise groups for each reader which resulted in 10 comparisons per reader. Thus, significant *p* value was calculated as 0.05/10 = 0.005.

All statistical computations were performed using IBM SPSS Statistics version 26.0.0.1 (IBM, Armonk, NY, USA).

## Results

### Patients

#### Training group

The patients in the training group were referred for clinically indicated PET-CT, due to known or suspected malignancy or infection. The five most common indications were lung cancer (n = 20), colorectal cancer (n = 11), lymphoma (n = 10), gynaecological cancer (n = 9) and symptoms indicating malignancy (n = 8). Fifty-four (54) % of the patients were female. Mean age was 61 years (SD 15 years, range 25–86 years). Mean height was 171 cm (SD 9.4 cm, range 154–197 cm), mean weight 74 kg (SD 14.8 kg, range 45–112 kg) and mean body mass index 25.4 (SD 4.6, range 16.9–36). Mean administered activity was 4.0 MBq/kg (SD 0.1, range 3.1–4.2 MBq/kg) and mean accumulation time 61 min (SD 3 min, range 56–69 min).

#### Test group

The 25 patients in the test group were referred for clinically indicated PET-CT, due to known or suspected malignancy: lung cancer (n = 13), colorectal cancer (n = 3), oesophageal cancer (n = 2), breast cancer, gynaecological cancer, head/neck cancer, testicular cancer, sarcoma, bile duct cancer and unclear bone finding (n = 1 each). Fifteen subjects were males and 10 females. Mean age was 59 years (SD, 14 years, range 24–81 years). Mean height was 173 cm (SD 9.5 cm, range 158–197 cm), mean weight 70 kg (SD 11.5 kg, range 44–92 kg) and mean body mass index 23.5 (SD 3.5, range 16.5–29.7). Mean administered activity was 4.0 MBq/kg (SD 0.1, range 3.8–4.3 MBq/kg) and mean accumulation time 62 min (SD 4 min, range 55–74 min).

**Fig. 3:**
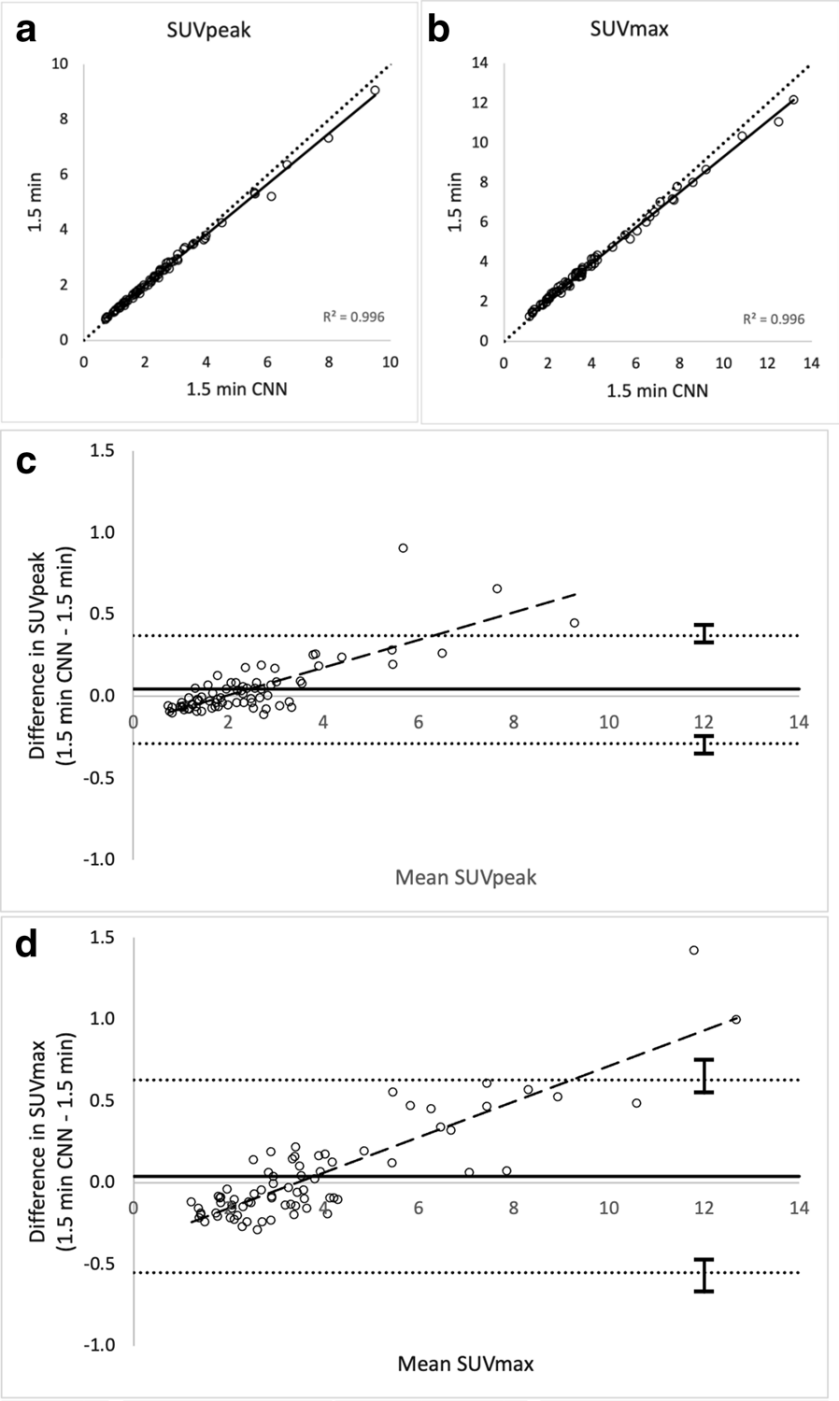
Agreements and correlations between 1.5 min CNN–1.5 min image sets for SUV_max_, and SUV_peak_. Pearson correlations (**a** and **b**; dotted line represents the line of identity and solid line shows the linear correlation). Bland–Altman plots (**c** and **d**). Solid horizontal line shows the mean of differences (0.04 and 0.04, respectively). Dotted lines represent 95% limits of agreement (0.37 to − 0.29 and 0.63 to − 0.55, respectively). Dashed line indicates trend. Error bars show 95% confidence limits calculated by exact method)

**Fig. 4:**
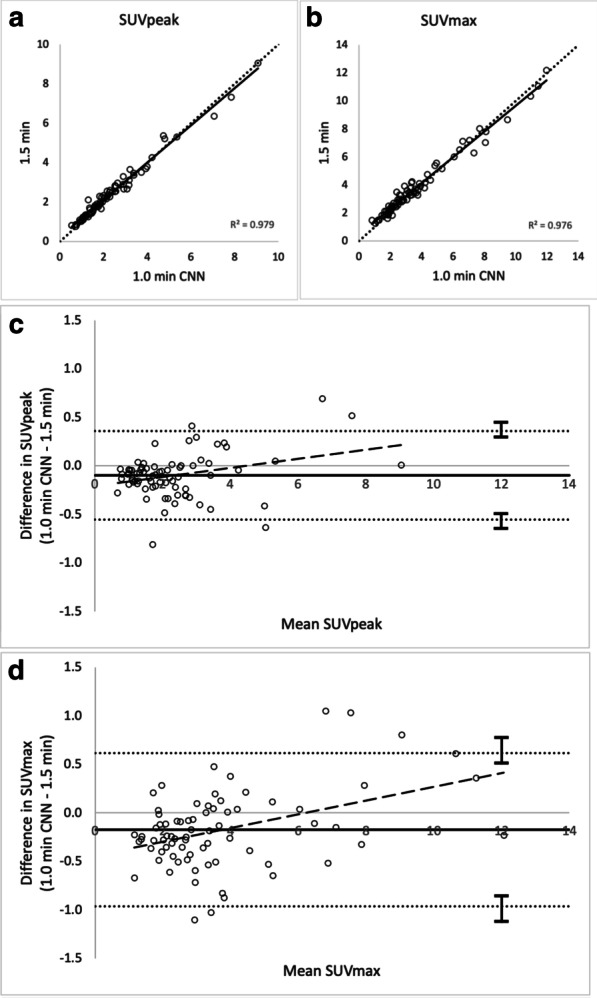
Agreements and correlations between 1.0 min CNN–1.5 min image sets for SUV_max_, SUV_peak_. Pearson correlations (**a** and **b**). Bland–Altman plots (**c** and **d**). Mean of differences were (− 0.10 and − 0.18, respectively). 95% limits of agreement were (0.36 to − 0.56 and 0.61 to − 0.97, respectively)

**Fig. 5:**
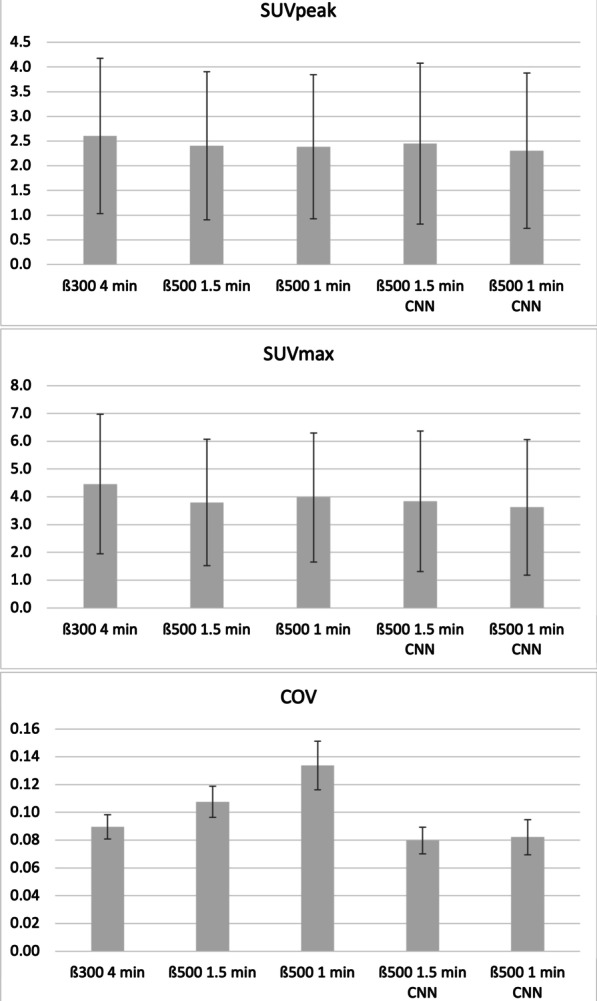
Comparisons of mean SUV_peak_, SUV_max_ and COV with error bars showing standard deviations for all image sets. SUV_peak_ mean values for both 1.5 min and 1.5 min CNN were 2.4. SUV_max_ mean values for both 1.5 min and 1.5 min CNN were 3.8. COV for 1.5 min CNN was less than the 1.5 min image set, indicating less noise

**Fig. 6:**
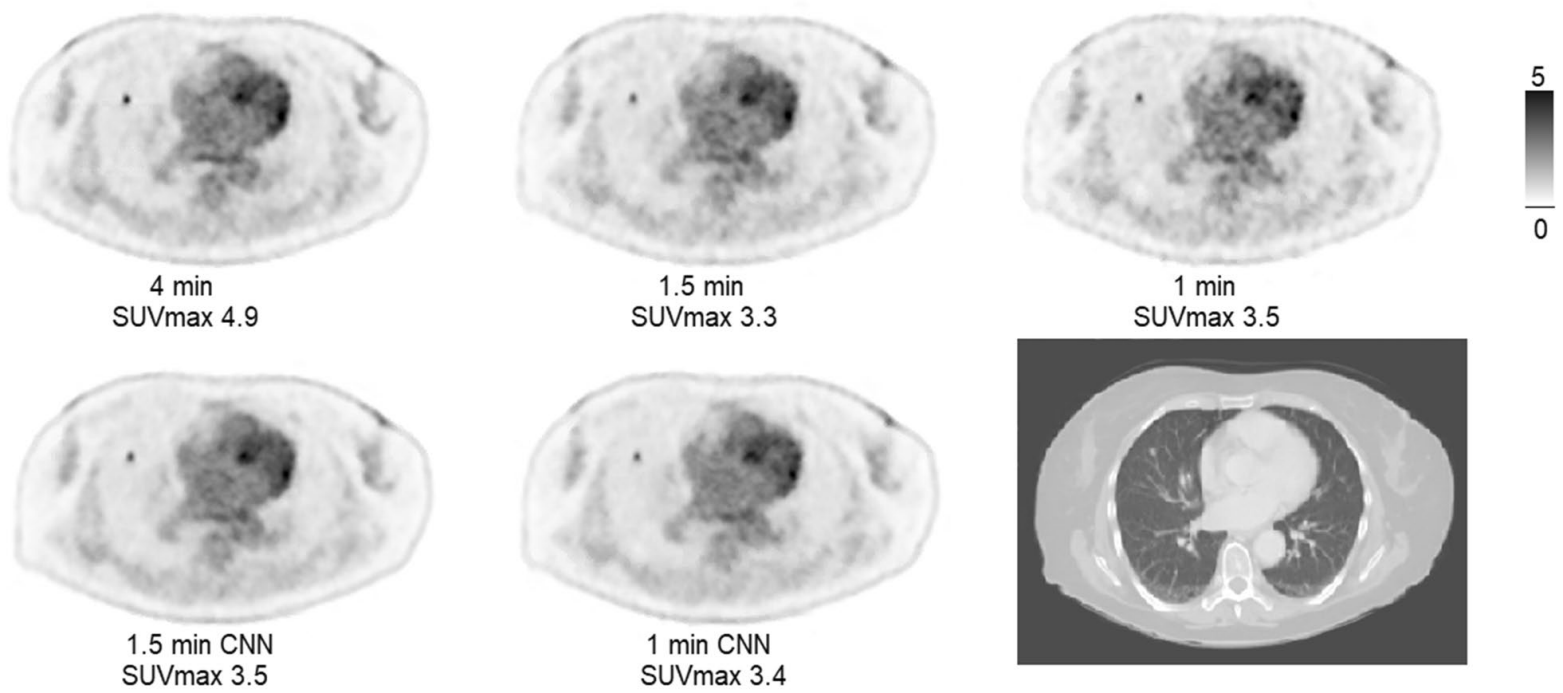
Transaxial PET images of all image sets and corresponding transaxial CT image. The lung metastasis in the right lung is readily detected regardless of reconstruction and the SUV_max_ in the metastasis is comparable for all 1 min and 1.5 min images

**Fig. 7:**
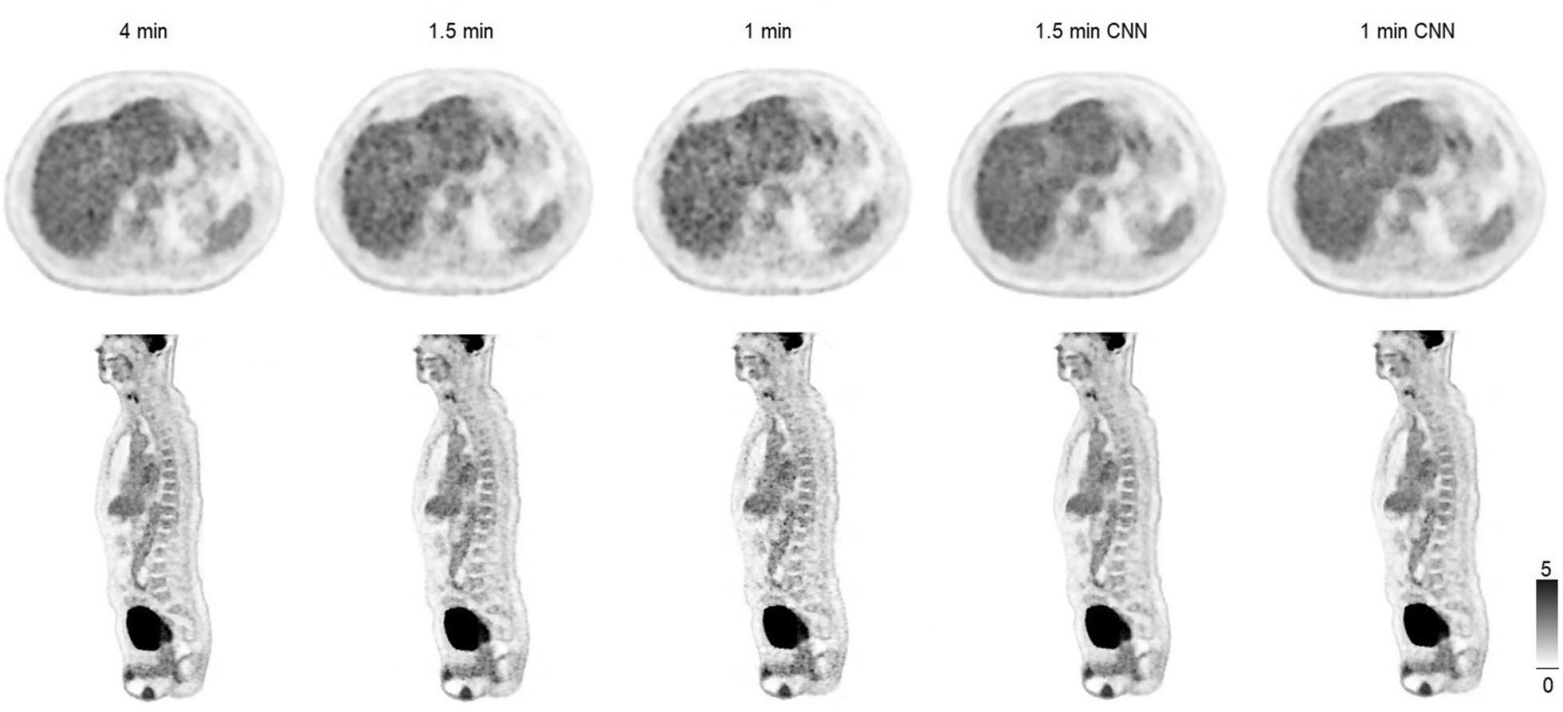
Transaxial PET images of the upper abdomen and midline sagittal images of the image sets in the test group. Noise in the liver increases as scan time per bed position is decreased, both corresponding CNN series have markedly less noise in the liver

Lesion SUV_max/peak_ are shown in scatter and Bland–Altman plots in Fig. 3a–d (1.5 min CNN vs. 1.5 min) and Fig. 4a–d (1.0 min CNN–1.5 min). Comparisons of mean SUV_peak_, SUV_max_ and COV across all series are shown in Fig. 5. Examples of the image sets are shown in Figs. 6 and 7.

### Quantitative analysis

The differences in the groups SUV_peak_, SUV_max_, and COV were statistically significant. A majority of the post-hoc comparisons showed significant differences for all quantitative metrics (SUV_peak_, SUV_max_ and COV). For SUV_peak_, the differences in the pairs 1.5 min–1.5 min CNN, 1.5 min–1 min and 1.5 min CNN–1 min, respectively, fell short of significance. For SUV_max_, the pairs 1.5 min–1.5 min CNN and 1.5 min CNN–1 min, respectively, fell short of significance. A graphical representation of the results is shown in Fig. 5 and statistical results in Table 3.

**Table 3:** Wilcoxon signed-ranks test of all pairwise image sets

	*P* values		
	SUV_peak_	SUV_max_	COV
4 min–1.5 min	< 0.001	< 0.001	< 0.001
4 min–1.5 min CNN	< 0.001	< 0.001	< 0.001
4 min–1 min	< 0.001	< 0.001	< 0.001
4 min–1 min CNN	< 0.001	< 0.001	< 0.001
1.5 min–1.5 min CNN	0.467	0.607	< 0.001
1.5 min–1 min	0.877	0.001	< 0.001
1.5 min–1 min CNN	< 0.001	< 0.001	< 0.001
1.5 min CNN–1 min	0.528	0.007	< 0.001
1.5 min CNN–1 min CNN	< 0.001	< 0.001	0.048
1 min–1 min CNN	< 0.001	< 0.001	< 0.001

Significant *p* value is < 0.005 after Bonferroni correction

### Qualitative analysis

Reader 2 evaluated all image sets in the test group and found no added or subtracted hotspots in any image set.

Mean and SD of noise and contrast score respectively for each image set of each reader are presented in Table 4. Noise-wise, 1.5 min CNN had the highest mean score for all readers; contrast-wise, reader 1 scored 1.5 min CNN the highest followed by 4 min, whereas reader 2 and 3 scored 4 min the highest followed by 1.5 min CNN. The standard 1 min acquisition got lowest scores from all readers both regarding noise and contrast. For all readers, 1.5 min CNN and 1 min CNN were consistently ranked higher than their corresponding 1.5 min and 1 min image set. Rankings of image sets for noise and contrast are shown in Table 5a, b.

**Table 4:** Reader score descriptives

Image set	Reader/category	N	Minimum	Maximum	Mean	SD
1.5 min	Reader 1 noise	25	1	3	2.5	0.65
	Reader 1 contrast	25	1	4	2.8	0.69
	Reader 2 noise	25	1	5	3.0	0.93
	Reader 2 contrast	25	1	5	3.1	0.93
	Reader 3 noise	25	2	4	3.0	0.58
	Reader 3 contrast	25	1	5	3.0	0.79
1.5 min CNN	Reader 1 noise	25	4	5	4.5	0.51
	Reader 1 contrast	25	2	5	4.3	0.80
	Reader 2 noise	25	3	5	4.4	0.58
	Reader 2 contrast	25	2	5	4.1	0.76
	Reader 3 noise	25	3	5	4.4	0.58
	Reader 3 contrast	25	3	5	3.9	0.81
1 min	Reader 1 noise	25	1	2	1.0	0.20
	Reader 1 contrast	25	1	3	2.0	0.61
	Reader 2 noise	25	1	3	1.7	0.85
	Reader 2 contrast	25	1	4	2.3	0.98
	Reader 3 noise	25	1	4	2.0	0.87
	Reader 3 contrast	25	1	4	2.5	0.82
1 min CNN	Reader 1 noise	25	2	4	3.2	0.85
	Reader 1 contrast	25	1	5	3.7	0.90
	Reader 2 noise	25	3	5	4.1	0.73
	Reader 2 contrast	25	2	5	3.7	0.74
	Reader 3 noise	25	3	5	4.1	0.70
	Reader 3 contrast	25	2	5	3.6	0.71
4 min	Reader 1 noise	25	3	5	3.8	0.80
	Reader 1 contrast	25	3	5	4.2	0.83
	Reader 2 noise	25	3	5	4.2	0.65
	Reader 2 contrast	25	3	5	4.3	0.68
	Reader 3 noise	25	3	5	4.2	0.55
	Reader 3 contrast	25	3	5	4.2	0.78

**Table 5:** Image sets ranked by mean noise and contrast score for all readers, 1 = best rank

	Reader 1	Reader 2	Reader 3
*(a) Noise rank*			
1	1.5 min CNN	1.5 min CNN	1.5 min CNN
2	4 min	4 min	4 min
3	1 min CNN	1 min CNN	1 min CNN
4	1.5 min	1.5 min	1.5 min
5	1 min	1 min	1 min
*(b) Contrast rank*			
1	1.5 min CNN	4 min	4 min
2	4 min	1.5 min CNN	1.5 min CNN
3	1 min CNN	1 min CNN	1 min CNN
4	1.5 min	1.5 min	1.5 min
5	1 min	1 min	1 min

Group wise analysis showed significant differences in scoring of the image sets for noise or contrast for any reader (*p* < 0.001). In the post-hoc pairwise analysis we found that there were significant differences in scoring for both noise and contrast for 1.5–1.5 min CNN across all comparisons. In the 1.5–1 min CNN comparisons, half of the mean rank scores were significantly different, detailed results for all pairwise comparisons are shown in Table 6.

**Table 6:** Mann Whitney U pairwise comparisons between image sets

Pairwise comparisons	*p* values					
	Reader 1 noise	Reader 1 contrast	Reader 2 noise	Reader 2 contrast	Reader 3 noise	Reader 1 contrast
4 min–1.5 min	< 0.001	< 0.001	< 0.001	< 0.001	< 0.001	< 0.001
4 min–1.5 min CNN	0.002	0.728	0.184	0.368	0.080	0.161
4 min–1 min	< 0.001	< 0.001	< 0.001	< 0.001	< 0.001	< 0.001
4 min–1 min CNN	0.015	0.031	0.716	0.009	0.706	0.005
1.5 min–1.5 min CNN	< 0.001	< 0.001	< 0.001	< 0.001	< 0.001	0.001
1.5 min–1 min	< 0.001	< 0.001	< 0.001	0.004	< 0.001	0.015
1.5 min–1 min CNN	0.009	< 0.001	< 0.001	0.013	< 0.001	0.009
1.5 min CNN–1 min	< 0.001	< 0.001	< 0.001	< 0.001	< 0.001	< 0.001
1.5 min CNN–1 min CNN	< 0.001	0.006	0.114	0.078	0.65	0.192
1 min–1 min CNN	< 0.001	< 0.001	< 0.001	< 0.001	< 0.001	< 0.001

Significant *p* value is < 0.005 after Bonferroni correction

## Discussion

The most important aspect when applying AI enhancement on medical images is that no disease specific information is subtracted or added, in the present study we have not found any evidence of this. The main goal was to elucidate if AI enhancement could help to improve image quality while preferably keeping a 1:1 ratio of SUV parameters compared to current clinical parameters. Improved image quality can be used for either providing the nuclear medicine physicians better images or to reduce scan time or administered activity. The rationale for not comparing with a post-filter such as Gaussian or bilateral filtering is due to evidence that BSREM intrinsic properties allows the omission of post-filtering [19].

The present study shows that the CNNs improved the 1 min and 1.5 min image sets qualitatively, respectively. The 1.5–1 min CNN comparison had higher mean rank by all readers for both noise and contrast, all significant without Bonferroni correction but 3 out of 6 comparisons fell just short of significance with the correction, but 1 min CNN had slightly lower mean SUV_max_ and SUV_peak_. We have not investigated if the difference is significant for the detection of disease or implicates assessments of follow-up examinations. Using the 1 min CNN image set would reduce scan time per bed position from 1.5 to 1 min in our county, which may decrease patient discomfort and movement artefacts. The 1.5 min CNN scored higher than 1 min CNN for both noise and contrast across all readers. Since the quantitative difference for SUV_max/peak_ between 1.5 and 1.5 min CNN fell short of significance, an introduction to the enhanced examination seems feasible in a clinical context, whereas it remains unclear if 1 min CNN can be used clinically due to the slightly reduced SUV_max/peak_ compared to the clinical 1.5 min image set. This could be elucidated in a future study where the two image sets are compared regarding diagnostic sensitivity.

We used 6 min images with β200 for training since this gives excellent images and was the longest feasible scan time for the patients to be able to keep up patient throughput in the department (where only one bed position was scanned with the long acquisition time). For the test group, we aimed for the best possible whole-body reference, and therefore used a slightly lower acquisition time (4 min) for all bed positions. [^18^F]FDG continues to accumulate in FDG-avid tumours long after 60 min [20]. If 6 min was used for all bed positions in the test group, then the latter positions may have had unacceptable biokinetics for the clinical evaluation of possible hotspots. Also, 6 min for all bed positions would be an uncomfortable long scanning time for the patient and not possible due to a demand of high patient throughput. The β values used for the different acquisition times were chosen based on previous work [7]. The settings used for the standard 1.5 min image are in line with the updated EANM Research Ltd (EARL) harmonization programme [21].

There are many papers which utilize both AI and non-AI methods to reduce noise in the PET image, some in conjunction with MRI, as a post-processing method (such as this study) or incorporated in the iterative reconstruction [22–26]. Other efforts in this field have been demonstrated by Xu et al. [27] where deep learning was used to reconstruct standard-dose PET from 200 × low-dose PET, which could increase the availability and reduce the radiation of radiopharmaceuticals. Cui et al. [28] used unsupervised deep learning to achieve denoising of PET images, their method employ the anatomical images from CT or MR and noisy PET images as inputs, omitting the need of large datasets of high-quality PET images which is not always easy to obtain. We have only found one study which investigated several AI enhancing techniques whilst aiming to keep quantitative accuracy in small lung nodules [22]. The study differs from ours in two major ways. Firstly, their methods aim to reproduce standard-dose PET from low-dose PET through AI methods while we are using a post-processing AI method. Secondly, our study aims to ensure that the CNN model does not alter SUV_max/peak_ in a way that is detrimental to the patient, such as affecting treatment response assessment. Detectability of lesions does not seem to be an issue in the present study (we did not find any added or subtracted hotspots), although it has not been investigated explicitly.

A continuation on Xu et al.’s discovery was exemplified by Chen et al., who used deep learning to synthesize ultra-low dose PET ([^18^F]florbetaben) with MRI data to predict standard-dose PET images and showed that both image quality metrics and accuracy of amyloid status was high [29]. A study by Schwyzer et al. demonstrated that deep neural networks were able to detect automatically lung cancer in ultra-low dose PET ([^18^F]FDG) [30]—one may speculate that AI technologies in conjunction will push nuclear medicine in a direction where ultra-low doses of PET-tracers are feasible, which translates into a wider application of the modality and increased frequency of examinations.

## Limitations

Subjective scoring, varying experience in PET assessment and image processing, and the fact that one author (reader 2) evaluated the examinations once before scoring may have had an impact on scoring results. A training/calibration session was held before individual scoring to reduce subjectivity. Although reader 2 was exposed to all the series side-by-side, these were blinded (image set properties not shown), and in the subsequent assessment which took place two weeks after, the image sets were shown one by one in random order to avoid bias.

## Conclusion

AI can enhance BSREM reconstructed [^18^F]FDG-PET examinations to reduce noise and increase contrast compared with standard images whilst keeping SUV_max/peak_ stability. There were significant differences in scoring between the 1.5 and 1.5 min CNN image sets in all comparisons, the latter had higher scores in noise and contrast. Furthermore, difference in SUV_max_ and SUV_peak_ fell short of significance for that pair. The improved image quality can potentially be used either to provide better images to the nuclear medicine physicians or to reduce acquisition time/administered activity.

## Data Availability

The datasets used and/or analysed during the current study are available from the corresponding author on reasonable request.

## Abbreviations

AI: Artificial intelligence
CNN: Convolutional neural network
COV: Coefficient of variation
CT: Computed tomography
FDG: [18F]-fluorodeoxyglucose
PET: Positron emission tomography
ROI: Region of interest
SD: Standard deviation
SUV: Standard uptake value
